# Systemic inflammation and biological aging in the Health and Retirement Study

**DOI:** 10.1007/s11357-023-00880-9

**Published:** 2023-07-27

**Authors:** Helen C. S. Meier, Colter Mitchell, Thomas Karadimas, Jessica D. Faul

**Affiliations:** Survey Research Center, Institute for Social Research, University of Michigan, 426 Thompson Street, Ann Arbor, MI 48106-1248 USA

**Keywords:** Inflammation, DNA methylation, Epigenetic clocks, Health and Retirement Study

## Abstract

Chronic, low-level systemic inflammation associated with aging, or inflammaging, is a risk factor for several chronic diseases and mortality. Using data from the Health and Retirement Study, we generated a continuous latent variable for systemic inflammation from seven measured indicators of inflammation and examined associations with another biomarker of biological aging, DNA methylation age acceleration measured by epigenetic clocks, and 4-year mortality (*N* = 3,113). We found that greater systemic inflammation was positively associated with DNA methylation age acceleration for 10 of the 13 epigenetic clocks, after adjustment for sociodemographics and chronic disease risk factors. The latent variable for systemic inflammation was associated with 4-year mortality independent of DNA methylation age acceleration and was a better predictor of 4-year mortality than any of the epigenetic clocks examined, as well as mortality risk factors, including obesity and multimorbidity. Inflammaging and DNA methylation age acceleration may represent different biological processes contributing to mortality risk. Leveraging multiple measured inflammation markers to capture inflammaging is important for biology of aging research.

**Supplementary Information:**

The online version contains supplementary material available at 10.1007/s11357-023-00880-9.

## Introduction

Inflammation is a key component of our immune system. Acute inflammatory responses to infection or injury are integral for maintaining homeostasis [1]. However, increased basal inflammatory activity is linked with aging and often characterized by higher levels of observed inflammatory cytokines such as C-reactive protein (CRP), interleukin-6 (IL-6), tumor growth factor-β (TGF-β), tumor necrosis factor-α (TNF-α), and tumor necrosis factor-II (sTNFR-II) [2–7]. This chronic, low-level systemic inflammation associated with aging in the absence of infection or injury (or “inflammaging”) is thought to be maladaptive and a risk factor for morbidity and mortality [8]. Indicators of inflammaging, including elevations in cytokines IL-6, CRP, and TNF-a, are associated with all-cause mortality, as well as with a number of age-related chronic diseases, including cardiovascular disease, diabetes mellitus, cancer, frailty, and sarcopenia [6, 9–14].

Epigenetic clocks, most often estimated from immune cells in blood, are DNA methylation (DNAm) signatures of chronological age or aging phenotypes [15–19]. Epigenetic age acceleration (DNAmAA) is a hypothesized indicator of underlying differences between “biological” age and “chronological” age. DNAmAA has been associated with age-related health outcomes, chronic diseases, cognitive functioning, frailty, and mortality [9, 15, 19–24].

Though inflammaging and DNAmAA are both typically immune cell-derived markers of aging, few studies have examined their relationship with each other [25–27]. Furthermore, low-grade chronic inflammation characteristic of inflammaging is the result of complex immune system interactions, including cascades and feedback loops [28]. To date, studies have examined individual cytokines with DNAmAA, but none has characterized systemic inflammation from multiple biomarkers simultaneously. To address this gap, we first generated a measure of systemic inflammation using a latent variable factor analysis. We estimated the underlying construct of systemic inflammation from seven observed inflammatory indicators. We then examined the association between the systemic inflammation latent variable and DNAmAA and assessed whether the systemic inflammation latent variable or DNAm age acceleration was a better predictor of 4-year mortality in a nationally representative sample of US adults over the age of 50.

## Methods

### Sample

The Health and Retirement Study (HRS) is a nationally representative sample of Americans over the age of 50. The HRS was approved by the University of Michigan Health Sciences/Behavioral Sciences Institutional Review Board. A subsample of individuals who participated in the Health and Retirement 2016 Venus Blood Study had DNA methylation assays completed (*N* = 4,104) [29]. Participants were excluded from the present analysis if they were not age eligible for HRS or had missing data on the outcomes or covariates resulting in a final sample of 3,311.

### Systemic inflammation

**Fig. 1:**
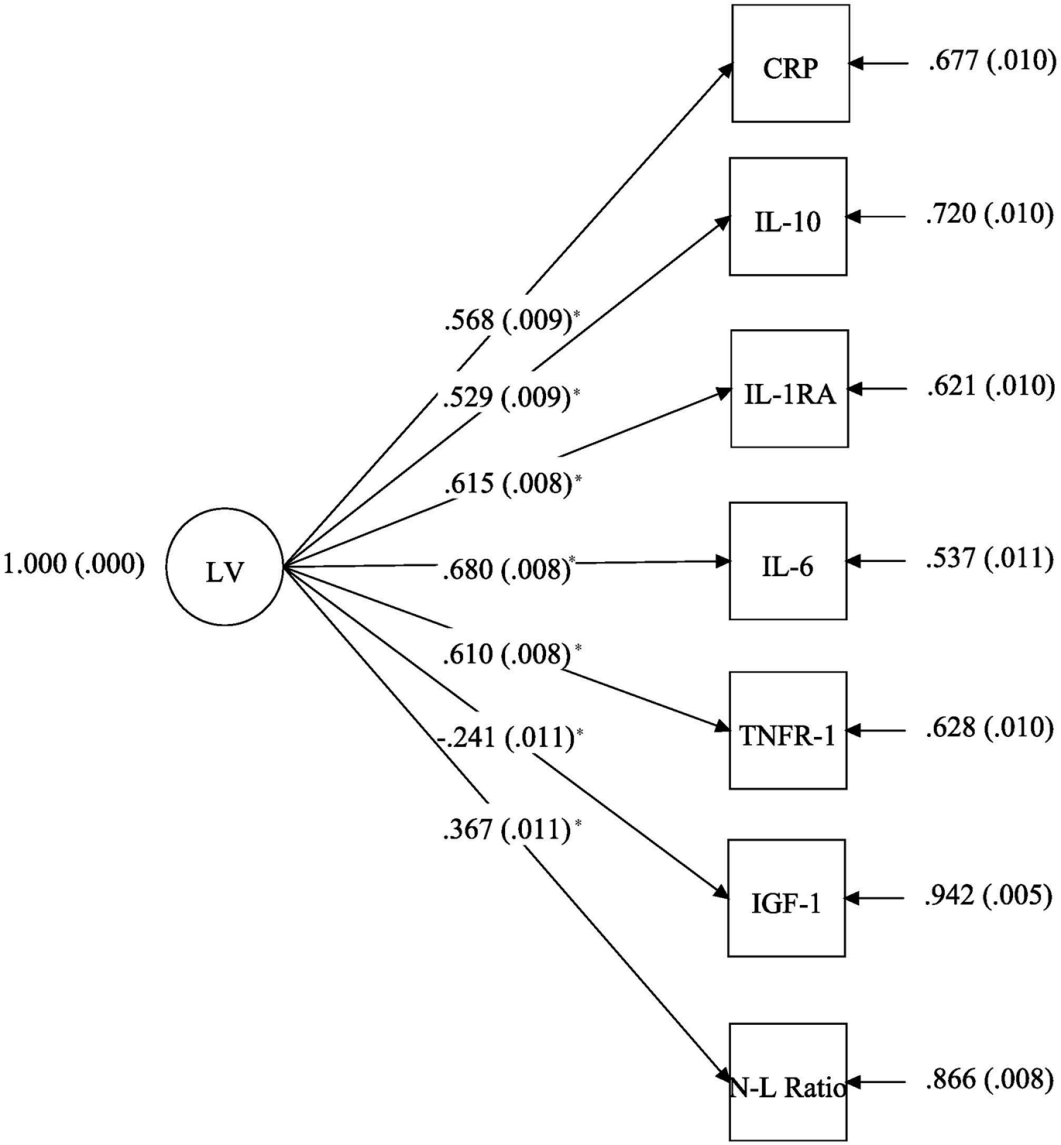
Standardized loading factors (coefficient from equation linking inflammatory measure to latent variable) and error terms (residual from equation) for measured indicators of the latent construct of systemic inflammation (CRP was referent, RMSEA: 0.072, CFI: 0.936). *Statistically significant < 0.001. Cytokines were log transformed. LV, latent variable; CPR, C-reactive protein; IL, interleukin; TNFR, tumor necrosis factor; IGF, insulin growth factor; N-L, neutrophil-to-lymphocyte

Venous blood was collected from participants by trained phlebotomists at home visits for the first time in 2016. Inflammatory cytokines, including high sensitive C-reactive protein (CRP), interleukin-6 (IL-6), interleukin-10 (IL-10), interleukin-1 receptor antagonist (IL-1RA), insulin-like growth factor 1 (IGF-1), and tumor necrosis factor (sTNFR-1), were assayed from serum at the University of Minnesota Advanced Research and Diagnostic Laboratory as described previously [29]. Neutrophil-to-lymphocyte ratio was derived from flow cytometry data from cryopreserved cells by dividing the percent of neutrophils by the percent of lymphocytes. A continuous latent variable representing overall systemic inflammation was created from the 6 log-transformed cytokine measurements, CRP (referent, loading factor fixed to 1), IL-6, IL-10, IL-1RA TNFR1, and IGF-1, and the neutrophil-to-lymphocyte ratio (RMSEA: 0.072, CFI: 0.936) using MPlus v8 (Muthén & Muthén, Los Angeles, CA). Standardized loading factors, error terms, and standard errors are provided in Fig. 1. Participant estimates for the systemic inflammation latent variable were exported from MPlus and merged with HRS data for analyses.

### DNA methylation measurement and calculation of epigenetic age

DNA methylation was measured from assays using the Infinium Methylation EPIC BeadChip. Assays were completed at the University of Minnesota as described previously [16]. Briefly, samples were randomized across plates by key demographic variables (i.e., age, cohort, sex, education, and race/ethnicity) with 39 pairs of blinded duplicates. Analysis of duplicate samples showed a correlation > 0.97 for all CpG sites. The *Minfi* package in R software, a suite of computational tools used to support preprocessing and quality control of Infinium Methylation EPIC BeadChip DNAm data [30], was used for HRS DNAm data preprocessing and quality control. Sex mismatched samples and any controls (cell lines, blinded duplicates) were dropped. *Minfi* flagged 3.4% of the methylation assay probes (*n* = 29,431 out of 866,091) for suboptimal performance (i.e., methylated + unmethylated DNA signal at a given position not different than the background signal level from a negative control) using a detection *P*-value threshold of 0.01. *Minfi* flagged 58 samples using a 5% cutoff for detection *P*-value failed samples, and these samples were removed from the final dataset. High-quality methylation data is available for 97.9% of the samples (*n* = 4,018).

Thirteen epigenetic clocks were calculated by HRS staff and made publicly available [16]. Details on the training dataset and which characteristic or phenotype (e.g., age and mortality) each epigenetic clock was trained on are available in HRS documentation [31]. Epigenetic age acceleration for each clock was generated by regressing age at 2016 on the epigenetic age and obtaining the residual. Epigenetic clock residuals were standardized with a mean of 0 and a standard deviation of 1 for cross-clock comparisons. Standardized residuals for each clock represent DNAmAA and were used in the present study.

### Covariates

Covariates included age at the 2016 study measured in years, sex assigned at birth (male or female), race/ethnicity (non-Hispanic White, non-Hispanic Black, or Hispanic), educational attainment (high school or less, some college, or more), marital status (married or not married), cytomegalovirus (CMV) seropositivity, multimorbidity, current smoking status (yes or no), obese (yes or no), heavy drinker (yes or no), and cell type proportions of the sample.

CMV seropositivity was categorized as positive or negative from IgG levels measured by the Roche e411 immunoassay analyzer (Roche Diagnostics Corporation, Indianapolis, IN). Reactive samples were categorized as positive, and borderline and non-reactive samples were categorized as negative. The sum of positive responses to whether a doctor has ever to the respondent that they have ever had a condition was generated from eight morbidities: high blood pressure, diabetes, cancer, lung disease, heart disease, stroke, psychiatric problems, and arthritis. Multimorbidity was defined as having 2 or more of the 8 conditions. Heavy drinking was defined as having 3 or more drinks on the days that a participant drank in the last 3 months. Cell proportions of the sample were estimated using flow cytometry data available from the HRS.

### Statistical analyses

To ensure population-level representation, HRS epigenetic sample weights were used in analyses to account for differential selection probabilities by race/ethnicity and birth cohort and to correct for differential non-response. Weighted means and frequencies of the sample were calculated. Weighted linear regression was used to estimate the association of systemic inflammation with each epigenetic clock age acceleration adjusting for age, sex, race/ethnicity, marital status, current smoking, and cell proportions of the sample (model 1). Model 2 additionally adjusted for CMV positivity, multimorbidity, heavy drinking, and obesity. The *R*^2^ for each model was reported.

A subset of commonly investigated epigenetic clocks, HorvathAA, HannumAA, LevineAA, GrimAgeAA, ZhangAA, and the Methylation Pace of Aging (MPOA; DunedinPoAm38), were used in subsequent analyses. Weighted logistic regression was used to estimate the association between systemic inflammation latent variable and 4-year mortality alone and adjusting for each DNAmAA clock. Mortality models controlled for age, gender, race/ethnicity, marital status, current smoking status, CMV positivity, multimorbidity, heavy drinking, obesity, and cell proportions of the sample. Area under the receiver operating characteristic (AUROC) estimates for key covariates, DNAmAA measures, and systemic inflammation latent variable were generated from the 4-year mortality models. All analyses were conducted in SAS v. 9.4 (SAS Institute, Inc., Cary, NC) using PROC SURVEY procedures and HRS epigenetic sample weights in accordance with HRS data documentation [31].

## Results

Table 1 contains the descriptive statistics for the sample. The study sample had an average age of 68.37 years and was 53.76% female and majority non-Hispanic White (80.70%). A majority of the sample had a high school education or less (62.99%), 58.75% were married, 11.14% were current smokers, and 10.07% reported heavy drinking. Less than half of the sample was classified as obese (43.96%), and only 20.23% reported 2 or more chronic conditions, while 62.29% were CMV positive. Of the 3,113 participants, 378 died between 2016 and 2020.

**Table 1:** Study participant characteristics (*N* = 3,311)

Characteristic		*N*	%
Age (mean, SE)		68.37	0.189
Gender	Male	1,398	46.24
	Female	1,913	53.76
Race/ethnicity	Non-Hispanic White	2,289	80.7
	Non-Hispanic Black	556	10.26
	Hispanic	466	9.04
Education	HS or less	2,294	62.99
	> HS	1,017	37.01
Marital status	No	1,423	41.25
	Yes	1,888	58.75
Current smoker	No	2,946	88.86
	Yes	365	11.14
Heavy drinker	No	3,009	89.93
	Yes	302	10.07
Obese	No	1,821	56.04
	Yes	1,490	43.96
Multimorbidity	0 or 1	2,531	79.77
	2 +	780	20.23
CMV positivity	No	1,017	37.71
	Yes	2,294	62.29
4-year mortality	No	2,933	90.06
	Yes	378	9.94

Correlations between the inflammation latent variable and DNAmAA measures are reported in Supplementary Table 1. Standardized associations between systemic inflammation latent variable and each DNAmAA clock are provided in Table 2. Statistically significant positive associations were found for HannumAA, LevineAA, SkinBloodAA, LinAA, Vidal-BraloAA, GrimAgeAA, ZhangAA, and MPOA in models adjusted for age, gender, race/ethnicity, marital status, current smoking, and sample cell proportions. After additional adjustment for CMV positivity, multimorbidity, heavy drinking, and obesity, systemic inflammation was no longer statistically significantly associated with HorvathAA. YangAA and BrocklandtAA were inversely associated with systemic inflammation in both models. The highest *R*^2^ was observed for GrimAgeAA, YangAA, ZhangAA, MPOA, HannumAA, and LevineAA, respectively. Model 2 was re-run with log CRP as the independent variable (Supplementary Table 2) to compare to results from the systemic latent variable models. The systemic inflammation latent variable effect sizes were larger than log CRP effect sizes, and *R*^2^ estimates were equivalent or larger with the latent variable as the independent variable.

**Table 2:** Associations between systemic inflammation latent variable and DNA methylation age acceleration measures in the Health and Retirement Study (*N* = 3,311)

	Model 1			Model 2		
DNAmAA	Standardized beta	*P*-value	*R*^2^	Standardized beta	*P*-value	*R*^2^
Horvath	0.10664	**0.0101**	0.02692	0.06768	0.1265	0.03130
Hannum	0.32452	** < 0.0001**	0.1394	0.32142	** < 0.0001**	0.14170
Levine	0.46613	** < 0.0001**	0.08807	0.46825	** < 0.0001**	0.09295
SkinBlood	0.15824	** < 0.0001**	0.02883	0.13787	**0.0015**	0.03173
Lin	0.2378	** < 0.0001**	0.03432	0.21768	** < 0.0001**	0.03568
Weidner	0.03865	0.3478	0.03142	0.03699	0.3984	0.03445
Vidal-Bralo	0.39044	** < 0.0001**	0.08783	0.38551	** < 0.0001**	0.09217
GrimAge	0.57485	** < 0.0001**	0.44150	0.55477	** < 0.0001**	0.45240
Yang	− 0.2940	** < 0.0001**	0.36370	− 0.3148	** < 0.0001**	0.39970
Zhang	0.55258	** < 0.0001**	0.34820	0.56209	** < 0.0001**	0.35520
Brocklandt	− 0.2263	** < 0.0001**	0.06544	− 0.2358	** < 0.0001**	0.06938
Garagnani	0.02738	0.5023	0.03176	0.01716	0.7002	0.03583
MPOA	0.63514	** < 0.0001**	0.29140	0.62973	** < 0.0001**	0.29680

Model 1: adjusted for age, gender, race/ethnicity, married, current smoking, and cell proportions

Model 2: model 1 covariates and CMV positivity, multimorbidity, heavy drink, and obesity

Bold: statistically significant at alpha = 0.05

**Fig. 2:**
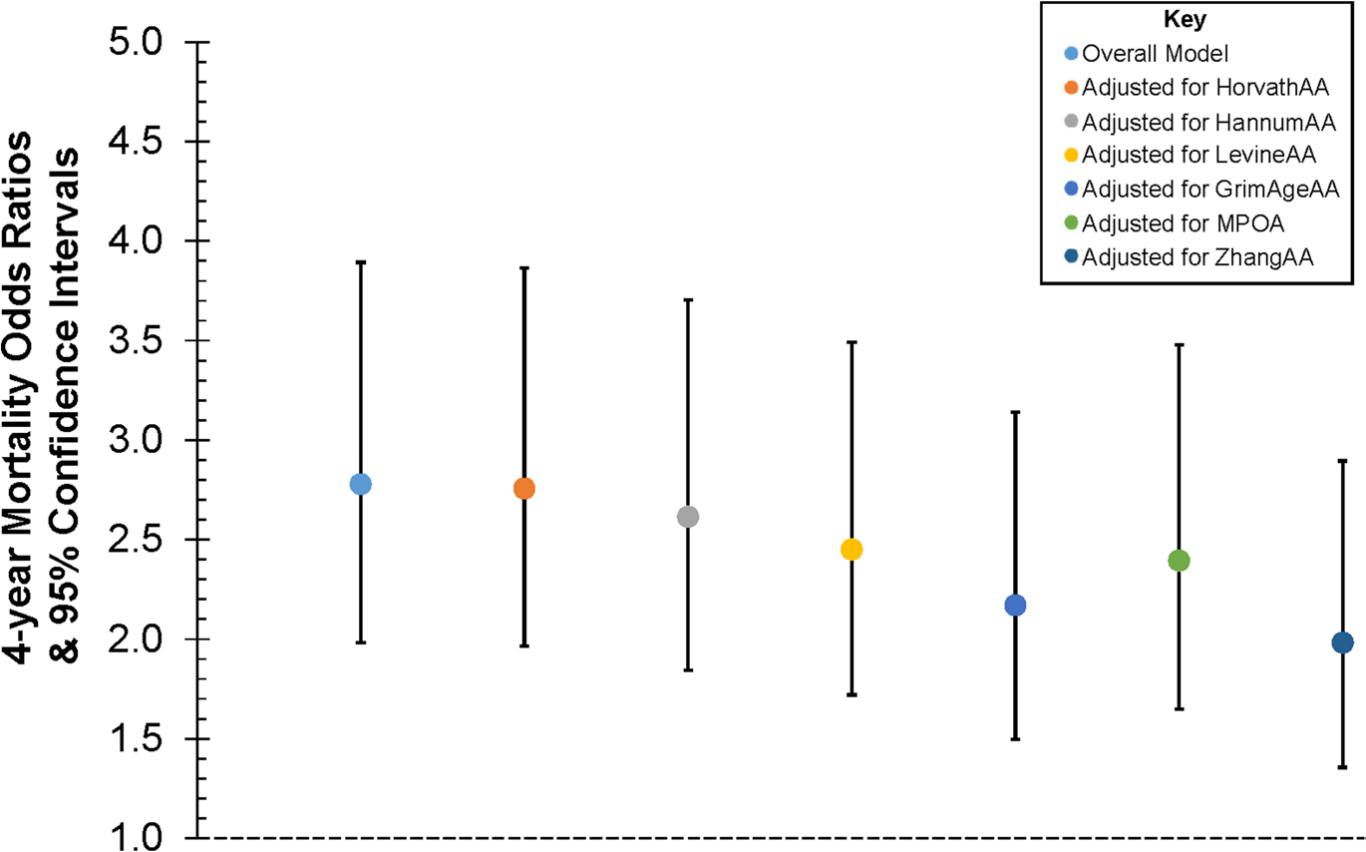
Overall association between the systemic inflammation latent variable and 4-year mortality adjusted for age, sex, race/ethnicity, education, marital status, multimorbidity, heavy drinking, obesity, smoking status, CMV positivity, and cell composition of the sample and then adjusted for DNAm age acceleration measures individually in the Health and Retirement Study (*N* = 3,113)

Table 3 shows adjusted odds ratios (ORs) and 95% confidence intervals (95% CIs) for the association between (1) systemic inflammation latent variable and 4-year mortality and (2) DNAmAA for 6 of the clocks and 4-year mortality for comparison. A one standard deviation increase in systemic inflammation was associated with 2.778 higher odds of 4-year mortality adjusting for covariates. For comparison, a one standard deviation increase in log CRP was associated with 1.42 higher odds of 4-year mortality (95% CI: 1.22, 1.64; data not shown). Figure 2 shows the association between systemic inflammation latent variable and 4-year mortality adjusted for age, sex, race/ethnicity, education, marital status, multimorbidity, heavy drinking, obesity, smoking status, CMV positivity, and cell composition of the sample and additionally adjusted for GrimAgeAA, YangAA, ZhangAA, MPOA, HannumAA, and LevineAA individually. After additionally adjusting for DNAmAA, the association between systemic inflammation latent variable and 4-year mortality was attenuated but remained statistically significant, and AUROC estimates (range 0.820–0.828) were slightly higher than the AUROC for the systemic inflammation latent variable model only (AUROC = 0.819). HannumAA, LevineAA, GrimAgeAA, ZhangAA, and MPOA were all associated with 4-year mortality adjusting for covariates (Table 3). Including systemic inflammation in the model attenuated 4-year mortality OR estimates for DNAmAA measures (Supplementary Table 3). HorvathAA was not associated with 4-year mortality in this sample. Individual factor AUROC estimates for 4-year mortality were calculated for age (0.74), systemic inflammation (0.68), multimorbidity (0.62), CMV positivity (0.53), and several DNAmAA measures: GrimAgeAA (0.66), ZhangAA (0.66), MPOA (0.63), LevineAA (0.62), HannumAA (0.58), and HorvathAA (0.53) (Supplementary Table 4).

**Table 3:** Independent associations between systemic inflammation latent variable (LV), DNA methylation age acceleration, and 4-year mortality in the Health and Retirement Study (*N* = 3,311)

Variable	4-year mortality OR	95% CI		Model AUROC
Inflammation LV	2.778	1.982	3.893	0.819
HorvathAA	1.101	0.938	1.294	0.816
HannumAA	1.327	1.159	1.521	0.804
LevineAA	1.437	1.260	1.639	0.809
GrimAgeAA	1.837	1.568	2.153	0.816
MPOA	1.488	1.280	1.731	0.809
ZhangAA	2.053	1.689	2.495	0.821

Adjusted for age, gender, race/ethnicity, education, marital status, multimorbidity, heavy drinking, obesity status, current smoking, CMV positivity, and cell composition of the sample (DNAmAA only)

## Discussion

Inflammaging and DNAmAA are two indicators of aging processes. In this study, we examined the association between systemic inflammation and 13 measures of DNAmAA and whether systemic inflammation or DNAmAA was more predictive of 4-year mortality in a nationally representative study of adults over age 50. We found that greater systemic inflammation was positively associated with DNAmAA for 10 of the 13 epigenetic clocks, after adjustment for sociodemographics and chronic disease risk factors, demonstrating remarkable consistency across “first”- and “second”-generation clocks. Prior research shows that epigenetic age acceleration was associated with individual measures of inflammation. Elevated levels of specific inflammatory cytokines and other inflammation-related molecules, including IL-6, CRP, TNF-a, IP-10, sTNFR2, IL-18, IL-18BP, and leptin, have been shown to be correlated with accelerated epigenetic aging [15, 25–27]. A recent study of 22 plasma-based inflammatory markers and four clocks (PhenoAge, GrimAge, DunedinPoAm, and Zhang) found associations between higher CRP and IL-6 and DNAmAA, but other indicators of inflammation were inconsistent [9]. The present study builds on this work by constructing a latent variable for systemic inflammation from multiple indicators instead of considering each inflammatory marker individually. Individual inflammatory marker measurements are indicators of a dynamic system, including complicated cascades and feedback loops; by using these individual measurements collectively, we were able to estimate the unmeasured, underlying construct of systemic inflammation to use in aging research. The systemic inflammation latent variable was associated with multiple epigenetic clocks in the HRS and exhibited larger estimated effect sizes and explained more variance than individual cytokines. The systemic latent variable was also a better predictor of 4-year mortality than any characteristic or clock other than chronological age.

We observed higher *R*^2^ values for inflammation with clocks that were trained on mortality, mitotic division, or biomarkers of aging, including GrimAgeAA, YangAA, ZhangAA, and MPOA, than for clocks trained on chronological age or phenotypic aging. MPOA used 18 biomarkers, including BMI, leukocyte telomere length, CRP, and white blood cell count, all of which either are inflammatory markers or associated with systemic inflammation [19]. Similarly, chronic inflammation is well known to be a risk factor for mortality [8]. In mouse models, inflammation was found to lead to abnormal methylation of polycomb target genes, which were used to develop the YangAA mitotic clock [32]. Thus, systemic inflammation is likely either an interdependent or co-occurring process with the factors these clocks were trained on, resulting in the inflammation latent variable explaining a higher proportion of the variance for these clocks.

While both systemic inflammation and DNAmAA measures were associated with 4-year mortality independently and after mutual adjustment, systemic inflammation was a better predictor of mortality in HRS than any of the epigenetic clocks, as well as other factors commonly associated with mortality, including multimorbidity, CMV positivity, and obesity. Only chronological age was a better predictor of 4-year mortality than systemic inflammation. This suggests that systemic inflammation and DNAmAA are related, but potentially representing different processes contributing to mortality. This finding is consistent with Cribb et al., who found that epigenetic aging and inflammaging were strongly and independently associated with mortality in a study of aging Australians [9]. Systemic inflammation may be a result of aging pathways involving cellular senescence, changes in adaptive immunity, or dysregulation of cellular processes [8] and for this study was estimated from biomarker measurement from venous blood, whereas the epigenetic clocks are DNAm signatures associated with age, mortality, and age-related phenotypes identified in other aging studies. It is possible that systemic inflammation was a better predictor of 4-year mortality than epigenetic age acceleration in this study because it was estimated directly from HRS participant biomarkers. Future work should confirm this finding in other representative studies of aging both with the USA and internationally.

This work has many strengths. This study was conducted in a large cohort representative of US adults over age 50 years. The HRS has wealth of information on participant sociodemographics, health behaviors, and biomarkers which were included as covariates, limiting unobserved confounding of associations. The inflammation latent variable was estimated from multiple indicators of inflammation, allowing for a more complete measurement of systemic inflammation than evaluating inflammatory markers individually. Sex differences in epigenetic age and immune function have been observed [16, 33, 34]. Exploratory analyses examining the association between systemic inflammation and DNAmAA stratified by sex were conducted but did not provide evidence of effect measure modification. This study also has limitations. The association between inflammation and DNAmAA was cross-sectional, limiting inference on the directionality of the relationship. However, the prediction of 4-year mortality by chronic inflammation and DNAmAA was a longitudinal analysis, providing insight into the relative utility of two biomarkers of mortality. The epigenetic clocks used in this study were trained in different study populations, different tissue types, and on numerous outcomes including chronological age, mortality, phenotypic aging, and age-related biomarkers. For example, PhenoAge was created using data from the InCHIANTI Study, which was a cohort of adults age 65 and older living in the Chianti geographic area of Tuscany, Italy [15]. The differences in study population characteristics between HRS and the InCHIANTI study could lead to measurement error in the estimation of PhenoAgeAA in HRS participants. However, the systemic inflammation-DNAmAA association was robust and observed for multiple clocks, lending support that this relationship is consistent across populations and body systems.

## Conclusion

We created a latent variable for systemic inflammation that correlated with other markers of biological aging and was a better predictor of 4-year mortality than all epigenetic age acceleration measures and participant characteristics except chronological age. We found that higher systemic inflammation was consistently associated with DNAm age acceleration across numerous epigenetic clocks in a US representative cohort of aging adults. Our results suggest that systemic inflammation and DNAm age acceleration were both risk factors for mortality, but that they may represent different biological processes contributing to mortality risk. Future work should replicate these findings in other US representative and international aging cohorts and examine longitudinal associations between systemic inflammation and DNAmAA to understand the directionality of the association.

### Supplementary Information

Below is the link to the electronic supplementary material.

Supplementary file1 (DOCX 19 KB)

## Data Availability

HRS data products are available at https://hrs.isr.umich.edu/data-products. Biomarker and sensitive health data are available from the public data portal after a supplemental agreement is signed.

## References

[1] Kennedy BK (2014). Geroscience: linking aging to chronic disease. Cell.

[2] Della Bella S (2007). Peripheral blood dendritic cells and monocytes are differently regulated in the elderly. Clin Immunol.

[3] Ballou SP (1996). Quantitative and qualitative alterations of acute-phase proteins in healthy elderly persons. Age Ageing.

[4] Bruunsgaard H (1999). A high plasma concentration of TNF-alpha is associated with dementia in centenarians. J Gerontol A Biol Sci Med Sci.

[5] Wei J (1992). Increase of plasma IL-6 concentration with age in healthy subjects. Life Sci.

[6] Bruunsgaard H, Pedersen M, Pedersen BK (2001). Aging and proinflammatory cytokines. Curr Opin Hematol.

[7] Frasca D (2016). The generation of memory B cells is maintained, but the antibody response is not, in the elderly after repeated influenza immunizations. Vaccine.

[8] Franceschi C, Campisi J (2014). Chronic inflammation (inflammaging) and its potential contribution to age-associated diseases. J Gerontol A Biol Sci Med Sci.

[9] Cribb L (2022). Inflammation and epigenetic aging are largely independent markers of biological aging and mortality. J Gerontol A Biol Sci Med Sci.

[10] Bruunsgaard H (2003). Predicting death from tumour necrosis factor-alpha and interleukin-6 in 80-year-old people. Clin Exp Immunol.

[11] Giovannini S (2011). Interleukin-6, C-reactive protein, and tumor necrosis factor-alpha as predictors of mortality in frail, community-living elderly individuals. J Am Geriatr Soc.

[12] Roubenoff R (2003). Cytokines, insulin-like growth factor 1, sarcopenia, and mortality in very old community-dwelling men and women: the Framingham Heart Study. Am J Med.

[13] Singh T, Newman AB (2011). Inflammatory markers in population studies of aging. Ageing Res Rev.

[14] Dugue PA (2022). Association of markers of inflammation, the kynurenine pathway and B vitamins with age and mortality, and a signature of inflammaging. J Gerontol A Biol Sci Med Sci.

[15] Levine ME (2018). An epigenetic biomarker of aging for lifespan and healthspan. Aging (Albany NY).

[16] Crimmins EM, et al. Associations of age, sex, race/ethnicity and education with 13 epigenetic clocks in a nationally representative US sample: the Health and Retirement Study. J Gerontol A Biol Sci Med Sci. 2021;76(6):1117–23. 10.1093/gerona/glab016. 10.1093/gerona/glab016 PMC8140049 33453106

[17] Horvath S (2013). DNA methylation age of human tissues and cell types. Genome Biol.

[18] Hannum G (2013). Genome-wide methylation profiles reveal quantitative views of human aging rates. Mol Cell.

[19] Belsky DW, et al. Quantification of the pace of biological aging in humans through a blood test, the DunedinPoAm DNA methylation algorithm. Elife. 2020;9:e54870. 10.7554/eLife.54870. 10.7554/eLife.54870 PMC7282814 32367804

[20] Maddock J (2020). DNA methylation age and physical and cognitive aging. J Gerontol A Biol Sci Med Sci.

[21] Lu AT (2019). DNA methylation GrimAge strongly predicts lifespan and healthspan. Aging (Albany NY).

[22] Chen BH (2016). DNA methylation-based measures of biological age: meta-analysis predicting time to death. Aging (Albany NY).

[23] Breitling LP (2016). Frailty is associated with the epigenetic clock but not with telomere length in a German cohort. Clin Epigenetics.

[24] Ammous F (2021). Epigenetic age acceleration is associated with cardiometabolic risk factors and clinical cardiovascular disease risk scores in African Americans. Clin Epigenetics.

[25] Irvin MR (2018). Metabolic and inflammatory biomarkers are associated with epigenetic aging acceleration estimates in the GOLDN study. Clin Epigenetics.

[26] Huang RC (2019). Epigenetic age acceleration in adolescence associates with BMI, inflammation, and risk score for middle age cardiovascular disease. J Clin Endocrinol Metab.

[27] Stevenson AJ (2018). Trajectories of inflammatory biomarkers over the eighth decade and their associations with immune cell profiles and epigenetic ageing. Clin Epigenetics.

[28] Ferrucci L, Fabbri E (2018). Inflammageing: chronic inflammation in ageing, cardiovascular disease, and frailty. Nat Rev Cardiol.

[29] Crimmins E, et al. Venus blood collection and assay protocol in the 2016 Health and Retirement Study 2016 Vensu Blood Study (VBS). 2017. Available at: https://hrsdata.isr.umich.edu/sites/default/files/documentation/data-descriptions/HRS2016VBSDD.pdf. Accessed Jan 2021.

[30] Aryee MJ (2014). Minfi: a flexible and comprehensive bioconductor package for the analysis of Infinium DNA methylation microarrays. Bioinformatics.

[31] Crimmins EM, et al. HRS epigenetic clocks report. 2020 [cited 2022 January 10]; Available from: https://hrsdata.isr.umich.edu/sites/default/files/documentation/data-descriptions/EPICLOCKS_DD.pdf. Accessed Dec 2021.

[32] Hahn MA (2008). Methylation of polycomb target genes in intestinal cancer is mediated by inflammation. Cancer Res.

[33] Kankaanpaa A (2022). Do epigenetic clocks provide explanations for sex differences in life span? A cross-sectional twin study. J Gerontol A Biol Sci Med Sci.

[34] Klein SL, Flanagan KL (2016). Sex differences in immune responses. Nat Rev Immunol.

