# Supplementary material for: Systemic inflammation and biological aging in the Health and Retirement Study
Source: GeroScience. 2023 Jul 27;45(6):3257–65. doi: 10.1007/s11357-023-00880-9 (PMC10643484; doi:10.1007/s11357-023-00880-9)
Supplement: Supplementary file 1 — Supplementary file1 (DOCX 19 KB) [file 11357_2023_880_MOESM1_ESM.docx]

**Supplementary Table 1.** Correlations between the systemic inflammation latent variable and DNAmAA for each clock in the Health and Retirement Study (N=4018)

|  | **Levine** | **Horvath** | **Hannum** | **GrimAge** | **MPOA** | **Skinblood** | **Lin** | **Yang** | **Zhang** | **Bocklandt** | **Weidner** | **Garagnani** | **VidalBralo** |
| --- | --- | --- | --- | --- | --- | --- | --- | --- | --- | --- | --- | --- | --- |
| **Inflammation** | 0.26432 | 0.0805 | 0.17086 | 0.32681 | 0.33741 | 0.09522 | 0.11379 | -0.10804 | 0.3075 | -0.09548 | 0.02197 | 0.039 | 0.17272 |
| p-value | <.0001 | <.0001 | <.0001 | <.0001 | <.0001 | <.0001 | <.0001 | <.0001 | <.0001 | <.0001 | 0.1639 | 0.0134 | <.0001 |

**Supplementary Table 2.** Adjusted associations between log C-reactive protein concentrations and DNA methylation age acceleration in the Health and Retirement Study (N=3,113).

| **DNAmAA** | **LogCRP**  **Standardized Beta** | **p-value** | **R^2^** |
| --- | --- | --- | --- |
| Horvath | 0.02878 | 0.1681 | 0.0314 |
| Hannum | 0.09503 | **<.0001** | 0.1273 |
| Levine | 0.1485 | **<.0001** | 0.06634 |
| SkinBlood | 0.03948 | 0.0709 | 0.02937 |
| Lin | 0.05298 | **0.0203** | 0.02806 |
| Weidner | -0.0125 | 0.5748 | 0.03446 |
| Vidal-Bralo | 0.13646 | **<.0001** | 0.0773 |
| GrimAge | 0.19766 | **<.0001** | 0.4227 |
| Yang | -0.1023 | **<.0001** | 0.3857 |
| Zhang | 0.15941 | **<.0001** | 0.3106 |
| Brocklandt | -0.0895 | **<.0001** | 0.06431 |
| Garagnani | -0.0028 | 0.9025 | 0.03582 |
| MPOA | 0.25686 | **<.0001** | 0.2714 |

Adjusted for age, sex, race/ethnicity, education, marital status, multimorbidity, drinking, obesity, current smoking, CMV positivity and cell composition of the sample. **Bold:** statistically significant at alpha = 0.05.

**Supplementary Table 3.** Odds ratios and 95% confidence intervals of for 4-year mortality mutually adjusted for inflammation latent variable (LV) and individual DNA methylation age acceleration clocks in the Health and Retirement Study (N=3,311)

|  | **4-year Mortality OR** | **95%CI** | |  | **4-year Mortality OR** | **95%CI** | |  | **MODEL ROC** |
| --- | --- | --- | --- | --- | --- | --- | --- | --- | --- |
| Inflammation LV | 2.756 | 1.965 | 3.864 | HorvathAA | 1.070 | 0.917 | 1.249 |  | 0.820 |
| Inflammation LV | 2.614 | 1.844 | 3.705 | HannumAA | 1.238 | 1.075 | 1.427 |  | 0.821 |
| Inflammation LV | 2.450 | 1.719 | 3.492 | LevineAA | 1.306 | 1.14 | 1.496 |  | 0.823 |
| Inflammation LV | 2.169 | 1.497 | 3.141 | GrimAgeAA | 1.575 | 1.325 | 1.872 |  | 0.826 |
| Inflammation LV | 2.394 | 1.648 | 3.478 | MPOA | 1.259 | 1.067 | 1.485 |  | 0.822 |
| Inflammation LV | 1.981 | 1.356 | 2.895 | ZhangAA | 1.800 | 1.458 | 2.221 |  | 0.828 |

Adjusted for age, sex, race/ethnicity, education, marital status, multimorbidity, drinking, obesity, current smoking, CMV positivity and cell composition of the sample.

**Supplementary Table 4.** Area under the curve receiver operating characteristic (AUROC) statistics for key covariates, DNAmAA and systemic inflammation predicting 4-year mortality

| **Variable** | **AUROC** |
| --- | --- |
| Chronologic Age | 0.7442 |
| Inflammatory latent variable | 0.6798 |
| GrimAA | 0.6603 |
| ZhangAA | 0.6583 |
| MPOA | 0.6282 |
| Multimorbidity | 0.6239 |
| LevineAA | 0.6174 |
| HannumAA | 0.5781 |
| HorvathAA | 0.5331 |
| CMV positivity | 0.5315 |
| SkinBloodAA | 0.5256 |
| Obesity | 0.4590 |

**Supplementary Table 5.** Mean and standard deviations for DNA methylation age and DNA methylation age acceleration (DNAmAA) in the Health and Retirement Study

| **Epigenetic Clock** | **DNAm Age Mean** | **Standard Deviation** | **DNAmAA (residual) Mean** | **Standard Deviation** |
| --- | --- | --- | --- | --- |
| Horvath | 65.68 | 9.57 | -0.08 | 6.47 |
| Hannum | 54.61 | 9.20 | -0.02 | 5.25 |
| Levine | 57.47 | 10.05 | -0.05 | 6.83 |
| SkinBlood | 69.64 | 8.80 | -0.01 | 4.43 |
| Lin | 58.39 | 11.06 | -0.06 | 7.83 |
| Weidner | 67.33 | 11.68 | 0.01 | 10.74 |
| Vidal-Bralo | 63.80 | 6.18 | 0.02 | 5.05 |
| GrimAge | 68.17 | 8.63 | 0.00 | 4.76 |
| Yang | 0.07 | 0.02 | 0.00 | 0.02 |
| Zhang | -1.08 | 0.46 | 0.00 | 0.44 |
| Bocklandt | 0.39 | 0.08 | 0.00 | 0.07 |
| Garagnani | 0.72 | 0.07 | 0.00 | 0.05 |
| MPOA | 1.07 | 0.09 | 1.07 | 0.09 |
